# β-arrestin 1 regulates β2-adrenergic receptor-mediated skeletal muscle hypertrophy and contractility

**DOI:** 10.1186/s13395-018-0184-8

**Published:** 2018-12-27

**Authors:** Jihee Kim, Chad A. Grotegut, James W. Wisler, Tianyu Li, Lan Mao, Minyong Chen, Wei Chen, Paul B. Rosenberg, Howard A. Rockman, Robert J. Lefkowitz

**Affiliations:** 1 Department of Medicine, Duke University Medical Center, Durham, NC USA; 2 Department of Obstetrics and Gynecology, Duke University Medical Center, Durham, NC USA; 3 Department of Medicine, Division of Cardiology and Duke Cardiovascular Physiology Core, Duke University Medical Center, Durham, NC USA; 4 Department of Cell Biology, Duke University Medical Center, Durham, NC USA; 5 Departments of Molecular Genetics and Microbiology, Duke University Medical Center, Durham, NC USA; 6 Department of Biochemistry, Duke University Medical Center, Durham, NC USA; 7 Howard Hughes Medical Institute, Duke University Medical Center, Durham, NC USA

**Keywords:** β-arrestin 1, β_2_AR, Clenbuterol, Hypertrophy, Contractility, Skeletal muscle

## Abstract

**Background:**

β_2_-adrenergic receptors (β_2_ARs) are the target of catecholamines and play fundamental roles in cardiovascular, pulmonary, and skeletal muscle physiology. An important action of β_2_AR stimulation on skeletal muscle is anabolic growth, which has led to the use of agonists such as clenbuterol by athletes to enhance muscle performance. While previous work has demonstrated that β_2_ARs can engage distinct signaling and functional cascades mediated by either G proteins or the multifunctional adaptor protein, β-arrestin, the precise role of β-arrestin in skeletal muscle physiology is not known. Here, we tested the hypothesis that agonist activation of the β_2_AR by clenbuterol would engage β-arrestin as a key transducer of anabolic skeletal muscle growth.

**Methods:**

The contractile force of isolated extensor digitorum longus muscle (EDL) and calcium signaling in isolated flexor digitorum brevis (FDB) fibers were examined from the wild-type (WT) and β-arrestin 1 knockout mice (βarr1KO) followed by chronic administration of clenbuterol (1 mg/kg/d). Hypertrophic responses including fiber composition and fiber size were examined by immunohistochemical imaging. We performed a targeted phosphoproteomic analysis on clenbuterol stimulated primary cultured myoblasts from WT and βarr1KO mice. Statistical significance was determined by using a two-way analysis with Sidak’s or Tukey’s multiple comparison test and the Student’s *t* test.

**Results:**

Chronic administration of clenbuterol to WT mice enhanced the contractile force of EDL muscle and calcium signaling in isolated FDB fibers. In contrast, when administered to βarr1KO mice, the effect of clenbuterol on contractile force and calcium influx was blunted. While clenbuterol-induced hypertrophic responses were observed in WT mice, this response was abrogated in mice lacking β-arrestin 1. In primary cultured myoblasts, clenbuterol-stimulated phosphorylation of multiple pro-hypertrophy proteins required the presence of β-arrestin 1.

**Conclusions:**

We have identified a previously unappreciated role for β-arrestin 1 in mediating β_2_AR-stimulated skeletal muscle growth and strength. We propose these findings could have important implications in the design of future pharmacologic agents aimed at reversing pathological conditions associated with skeletal muscle wasting.

**Electronic supplementary material:**

The online version of this article (10.1186/s13395-018-0184-8) contains supplementary material, which is available to authorized users.

## Backgrounds

G protein-coupled receptors (GPCRs) are the largest family of receptors and are fundamental in regulating physiological processes under normal and pathological conditions [1]. Among them, β-adrenergic receptors (βARs) play important roles in cardiovascular, pulmonary, and skeletal muscle physiology [2, 3]. Within the skeletal muscle, β_2_ARs are the dominant βAR subtype [4, 5]. In response to the endogenous catecholamine epinephrine, β_2_ARs in skeletal muscle activate a portfolio of signaling pathways that lead to anabolic and hypertrophic cellular growth [6]. The hypertrophic effect of β_2_AR-mediated signaling in skeletal muscle is associated with enhanced gene transcription and protein translation that lead to increased expression of skeletal muscle contractile proteins and decreased myofibrillar proteolysis [7]. Following myotoxic injury, β_2_AR stimulation can hasten the structural and functional recovery of regenerating skeletal muscle, which has led to the use of synthetic β_2_AR agonists to prevent or even reverse age and disease-related skeletal muscle weakness and wasting [8–10].

Agonist stimulation of β_2_ARs activates cellular signaling primarily through the G protein-mediated generation of intracellular second messengers such as cyclic adenosine monophosphate (cAMP), by the effector enzyme adenylyl cyclase [11, 12]. Following agonist binding, β_2_ARs undergo rapid desensitization through the sequential action of receptor phosphorylation by G protein-coupled receptor kinases (GRKs) and recruitment of the multifunctional adapter protein, β-arrestin [13, 14]. β-arrestins are ubiquitously expressed and as GPCR scaffold proteins are essential for processes that initiate receptor desensitization, internalization, and ubiquitination [14]. β-arrestins can also function as signal transducers to initiate signaling cascades independent of, or collaboratively with, G proteins thus markedly expanding the portfolio of cellular signaling downstream of GPCR activation [15]. Highlighting the importance of β-arrestins as signal transducers, recent studies using knockout mice have demonstrated critical functions of β-arrestins in physiological processes such as chemotaxis [16–18], the Frank-Starling force [19], and pathological conditions such as myelofibrosis [20], pulmonary fibrosis [21], and asthma [22, 23].

Based on the regulatory roles of β-arrestins in GPCR signaling, we hypothesized that the enhancement of skeletal muscle function by β_2_AR stimulation might be mediated by β-arrestin 1, which is the dominant β-arrestin isoform in skeletal muscle. In this study, we investigated the contractile properties of skeletal muscle fibers after chronic administration of the selective β_2_AR agonist clenbuterol in wild-type (WT) and β-arrestin 1 knockout (βarr1KO) mice. By assessing skeletal muscle fiber size, muscle composition, and contractile properties, we identified a fundamental role for β-arrestin 1 in β_2_AR-mediated skeletal muscle hypertrophy and further elucidated the molecular mechanism for the action of clenbuterol in isolated myoblasts.

## Methods

### Animal

All animal experimental protocols were approved by the Institutional Animal Care and Use Committee at Duke University Medical Center and were performed in accordance with the standards established by the US Animal Welfare Acts.

### Generation of β-arr1KO utilizing LoxP-Cre recombineering

β-arr1flox/flox mice were generated using recombineering techniques as previously described for the generation of β-arrestin 2 flox/flox mice [24]. All reagents (plasmids: PL253, PL451, PL452; and bacterial strains: SW102, SW105, and SW106) were obtained from NCI-Frederick Cancer Research and Development Center. A 17.3 kb fragment of mouse genomic DNA containing *βarr1* (*arrb1*) was retrieved from the mouse genomic DNA BAC library (BACPAC Resource Center, Children’s Hospital Oakland Research Institute, Oakland, CA) to PL253 plasmid, which contains an Mc1-driven Thymidine Kinase (TK) cassette for negative selection in embryonic stem (ES) cells. Two loxP cassettes were inserted into *arrb1* gene flanking coding Exon 2. The vector also contains FRT-Neo-FRT cassette inserted downstream of the short homologous sequence. ES cell targeting and generation of chimeric mice with *arrb1* loxP allele were performed by the Duke Transgenic Mouse Facility. The NotI-linearized targeting vector was inserted into ES cells derived from 129S6/SvEvTac mouse. Positive ES clones were injected into mouse blastocysts to produce chimeric mice, which were then crossed with C57Bl/6J mice (The Jackson Laboratory, Bar Harbor, ME, USA) to allow germline transmission and produce heterozygote mice harboring *arrb1* loxP/FRT-Neo-FRT allele. In vivo excision of the FRT-Neo-FRT cassette done by crossing the heterozygote mice with a transgenic mouse expressing FLPe recombinase (B6SJLTg (ACTFLPe) 9205Dym/J; The Jackson Laboratory, Bar Harbor, ME, USA) led to the establishment of heterozygote mice harboring *arrb1-*loxP allele (*arrb1*wt/flox). Intercrossing of these heterozygote mice (*arrb1*wt/flox) resulted in homozygous βarr1flox/flox mice, and these animals were subsequently backcrossed into a C57/B6 genetic background for seven generations.

The βarr1flox/flox mice were then crossed with β-actin (ACTB)-Cre transgenic mice (Jackson Laboratories, Bar Harbor, ME, USA) with ubiquitously expressed Cre recombinase under the control of the β-actin promoter. After identification of mice with the floxed allele and cre transgene, they were subsequently backcrossed into a C57/B6 genetic background for 7 generations.

### Immunoblotting

Tissue extracts were prepared using glycerol lysis buffer [25]. Equal amounts of tissue lysates (20–100 μg of total protein) were separated on 10% Tris-glycine polyacrylamide gels (Invitrogen) and were transferred to nitrocellulose membranes for immunoblotting. β-arrestin 1 and GAPDH were detected by immunoblotting with rabbit polyclonal anti-β-arrestin (A1CT, 1:3000) [25] and monoclonal anti-GAPDH (1:5000) antibodies, respectively. Chemiluminescent detection was performed using the SuperSignal West Pico reagent (Pierce).

### Chronic drug delivery using isotonic pump

Eight to 12-week-old mice of either sex were used. After anesthetizing with isoflurane, a subcutaneous osmotic pump (Alzet 2004 and Alzet 2002: Durect, Cupertino, CA, USA), containing either vehicle (10% dimethyl sulfoxide and 0.3 mM ascorbic acid) or clenbuterol (1 mg/kg/day) dissolved in vehicle, was placed in the tissue immediately lateral to the spine on the posterior of the animal. Muscle contraction was measured after 2 weeks of vehicle or clenbuterol treatment while muscle hypertrophy was examined after 4 weeks. Chemicals used were purchased from Sigma-Aldrich (St. Louis, MO, USA).

### Measurement of the contractile properties of extensor digitorum longus (EDL)

Animals were anesthetized using ketamine/xylazine (100 and 2.5 mg/kg, respectively), each EDL was then dissected free, and a 6–0 silk suture was secured to each EDL tendon. The isolated EDL was then suspended in a Radnoti (Radnoti LLC, Montrovia, CA, USA) 10 ml tissue organ bath between stainless steel wire hooks connected to a Radnoti isometric force transducer and a glass hook inside the organ bath that served as an anchor. The bath was filled with modified Krebs buffer (118 mM NaCl, 4.8 M KCl, 1.2 mM MgSO_4_, 1.2 mM KH_2_PO_4_, 2.5 mM CaCl_2_, 25 mM NaHCO_3_, and 11 mM glucose, pH 7.4), maintained at 25 °C, and constantly bubbled with a premixed gas consisting of 20% O_2_, 5% CO_2_, and balance N_2_. All muscle contraction responses were recorded using LabChart7 (ADInstruments, Colorado Springs, CO) connected to a PowerLab 4/30 (ADInstruments). Using a Grass Technologies (Grass Technologies, Warwick, RI, USA) S88H square pulse stimulator, the optimal EDL length was found by gradually increasing the tension on the EDL, and applying a 0.5 ms square pulse up to supramaximal stimulus (40 V), until no further increase in twitch tension was observed [26]. The amplitude from the isolated EDL at optimal length was recorded using LabChart. The twitch response was calculated as the twitch amplitude at supramaximal stimulus (40 V) with a 0.5 ms square pulse. Tetanus responses were compared between groups as the response seen at 160 Hz. The force-frequency relationship was determined by measuring the maximal amplitude at each train stimulus (300 ms) with supramaximal voltage (40 V) at 30 Hz, 60 Hz, 100 Hz, 140 Hz, and 160 Hz trains with a 3-min rest between each train. The amplitude at each frequency and for each treatment group was normalized by cross-sectional area (CSA). To measure fatigue response, a 100 Hz stimulus for 300 ms at supramaximal voltage was applied every 3 s for 10 min. Time to fatigue was determined to be the time the EDL took to contract at 50% of the amplitude obtained at the initial 100 Hz stimulus. Curves were compared using a two-way ANOVA with Tukey’s multiple comparison test. For the statistical comparison of two conditions, the Student’s *t* test was used (Prism).

### Calcium imaging of flexor digitorum brevis (FDB) fibers

FDB fibers were isolated using 0.2% (wt/vol) collagenase type I solution from the mice implanted with either vehicle or clenbuterol and maintained in Dulbecco’s Modified Eagle Medium (DMEM) with 10% horse serum [27]. To monitor cellular Ca^2+^ concentration, myofibers were loaded with Fura-4-acetoxymethyl ester (1 μM; Molecular Probes/Invitrogen) for 30 min at room temperature, washed, and incubated for 30 min in dye-free buffer. Incubations were carried out in Opti-MEM with 10% horse serum and washes were carried out in Ringer solution (140 mM NaCl, 2.8 mM KCl, 2 mM CaCl_2_, 2 mM MgCl_2_, 10 mM glucose, and 10 mM HEPES, pH 7.4). Electrical field stimulation of myofibers was delivered via a 35-mm dish insert with a pair of platinum electrodes lining the perfusion trough (RC-37FS; Warner Instruments), and the electrical stimulus was generated by an A310 Accupulser (World Precision Instruments) and an A385 Stimulus Isolator (World Precision Instruments) connected to the dish insert. Bursts of stimulation, each of which is called a “stimulus” hereinafter, consisted of 1-ms current pulses (100 mA) applied at 50 Hz for the indicated stimulus duration. The stimulus-response curve was generated by applying such stimuli at a range of stimulus durations (100 ms to 5 s), with a single test stimulus applied every 50 s. Curves were compared using a two-way repeated measures ANOVA with Sidak’s multiple comparison test. To monitor basal Ca^2+^concentration, FDB myofibers were loaded with Fura-2-acetoxymethyl ester (1 μM; Molecular Probes/Invitrogen) for 30 min at room temperature, washed, and incubated for 30 min in dye-free buffer. Intracellular calcium levels were quantitated by the Fura-2 excitation ratio at 340 and 380 nm on an epifluorescence microscope. The mean response for each treatment group was compared using a two-way ANOVA and the Student’s *t* test (Prism).

### Immunohistochemistry

Muscles were isolated after 4 weeks of drug delivery and mounted in O.C.T. (optimal cutting embedding medium) with gum tragacanth (Sigma, at a 4:1 ratio), and flash frozen in an isopentane bath suspended in liquid nitrogen. Cross sections of muscle were cut from the belly of the muscle at a thickness of 10 μm. Sections were fixed with ice-cold acetone for 10 min. For myofiber counts and cell size determination, a simple multicolor immunofluorescence procedure [28] was performed with primary antibodies against MHC I (BA-F8, 1:250 dilution), MHC IIa (SC-71, 2F7), MHC IIb (BF-F3), and polyclonal dystrophin (1:1000 dilution). Staining was visualized simultaneously using Alexa Fluor 488 anti-mouse IgG2a (BF-F3, MHC IIb), Alexa Fluor 568 anti-mouse IgG1 (SC-71, MHC IIa), Alexa Fluor 647 anti-mouse IgG2b (BA-F8, MHC I), and Alexa Fluor 405 anti-rabbit (dystrophin). Primary antibodies (BF-F3, SC-71, and BA-F8) were purchased from the Developmental Studies Hybridoma Bank (University of Iowa), anti-dystrophin antibody (Sigma-Aldrich), and secondary antibodies were purchased from Invitrogen. All sections were mounted in Vectashield (Vector labs). Slides were visualized with a Zeiss LSM510 laser scanning microscope (3i) using conventional wide field fluorescence microscopy. Individual images were taken across the entire cross-section and assembled into a composite montage image with Slidebook 6 program (3i). For fiber type analysis, all fibers within the entire muscle/cross-section were examined in a blind manner. Fiber CSA was measured for each fiber type by outlining at least 40% of all fibers within a muscle/cross-section using Image J program (NIH). Fiber type percentages and fiber CSA data are reported as group means ± SEM based on individual animal. The mean response for each treatment group was compared using a one-way ANOVA and the Student’s *t* test (Prism).

### Primary myoblast preparation

Primary myoblasts were isolated from WT and βarr1KO mice by collagenase digestion using a previously described protocol [29]. The hindlimbs were removed from 8-month-old mice and the bones and tendons were dissected away. The muscle was minced into a coarse slurry using razor blades in Hanks balanced salt solution (Gibco) with 1% glutamine and 1% of penicillin/streptomycin. Cells were enzymatically dissociated by 0.2% of collagenase II (Worthington) supplemented with 0.25% trypsin (Gibco) at 37 °C for 30–45 min and then passed through 100 µm cell stainer (Falcon). The filtrate was centrifuged at 3000 rpm for 5 min, and the pellet was resuspended in DMEM supplemented with 10% horse serum, 1% glutamine, and 1% penicillin/streptomycin. Cell suspension was subjected to Percoll gradient (70% and 40%) in a 15 ml conical tube and separated by centrifugation at 2500 rpm for 20 min at room temperature. Myoblasts were collected from 70% and 40% Percoll interface and transferred to fresh media plated on collagen-coated dishes.

### Phospho-antibody array analysis

The phospho-antibody array analysis was performed using the Proteome Profiler Human Phospho-Kinase Array Kit ARY003 (R&D Systems) according to the manufacturer’s instructions. Briefly, primary cultured myoblasts were serum-starved for 24 h and then stimulated with 10 μM clenbuterol for 5 min. Cells were lysed with Lysis Buffer 6 (R&D Systems) and agitated for 30 min at 4 °C. Cell lysates were clarified by microcentrifugation at 14,000×*g* for 5 min, and the supernatants were subjected to protein assay. Pre-blocked nitrocellulose membranes of the Human Phospho-Kinase Array were incubated with approximately 500 μg of cellular extract overnight at 4 °C on a rocking platform. The membranes were washed three times with 1× Wash Buffer (R&D Systems) to remove unbound proteins and then were incubated with a mixture of biotinylated detection antibodies and streptavidin-HRP antibodies (R&D Systems). Chemiluminescent detection reagents were applied to detect spot densities. Array images were analyzed using the GeneTools image analysis software (Syngene). Every spot was subtracted by the average background level from negative control spots and normalized by the density levels of its own positive control spots to validate the membranes from four different conditions. The averaged density of duplicated spots representing each phosphorylated kinase protein was determined and used for the relative changes in phosphorylated kinase proteins. The phospho-antibody array experiment was repeated at least three times. Statistical analysis was performed using a two-way ANOVA (PRISM Software) with Sidak’s multiple comparison test. For statistical comparison of two conditions, the Student’s *t* test was used (Prism).

### Statistical analyses

Data are presented as mean ± SEM. Statistical significance in each time point of kinetic graphs was determined by using a two-way analysis with Sidak’s or Tukey’s multiple comparison tests. The mean response for each treatment group was compared using a two-way ANOVA (prism) to check for the drug or strain effects. For the statistical comparison of two conditions, the Student’s *t* test was used. The level of significance is indicated as follows: ***P < 0.001, **P < 0.01, *P < 0.05.

## Results

### β-arrestin 1 is required for clenbuterol-mediated enhanced contractility of skeletal muscle

**Fig. 1:**
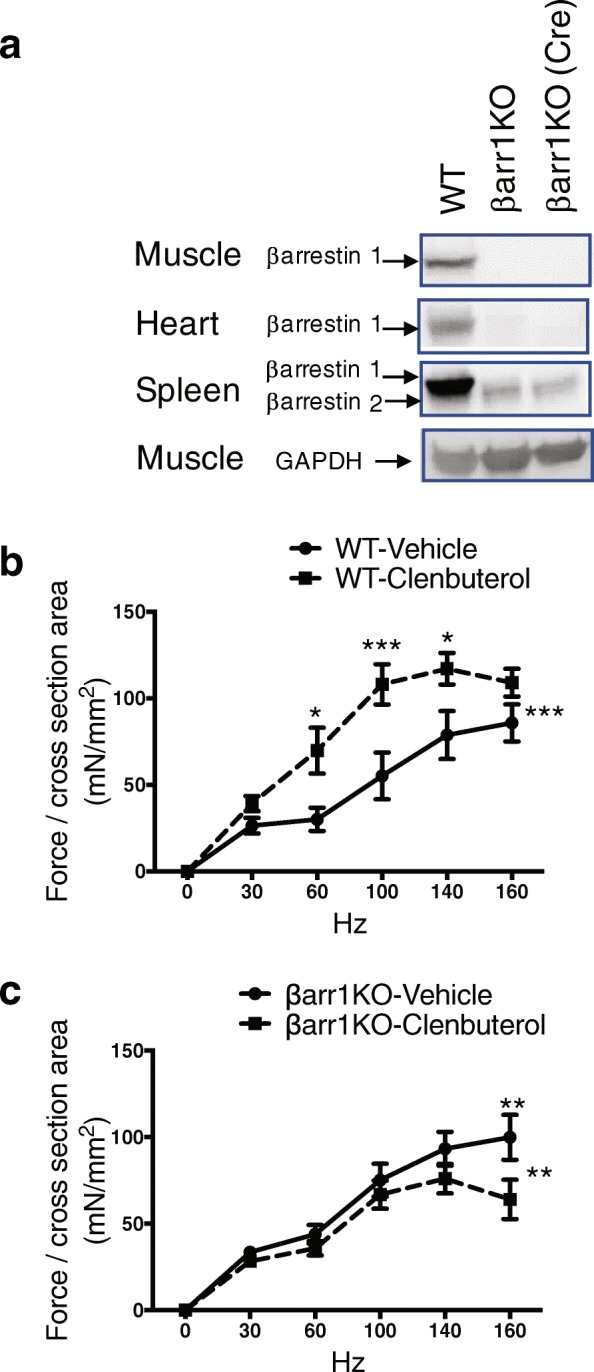
β-arrestin 1 is required for clenbuterol-mediated enhanced contractility of skeletal muscle. **a** Tissue lysates from muscle, heart, spleen of WT and βarr1KO mice subjected to measure β-arrestin 1 expression by immunoblotting using anti-β-arrestin 1 antibody (A1ct). Equal amounts of tissue lysates loaded were probed by immunoblotting using anti-GAPDH antibody. **b**, **c** Force-frequency curves for EDL muscles after 2 weeks of vehicle or clenbuterol (1 mg/kg/day) treatment from WT (**b**) and βarr1KO (**c**). Data represent the mean ± SEM from six to eight independent experiments. Statistical comparison of the curves was performed by using a two-way ANOVA with Tukey’s multiple comparison test (*, *P* < 0.05; **, *P* < 0.01; ***, *P* < 0.001)

β-arrestin 1 is the dominant β-arrestin isoform in skeletal muscle (Fig. 1a). To assess whether β-arrestin 1 mediates isometric tetanic force in response to clenbuterol, we utilized two mouse lines lacking β-arrestin 1 expression globally: βarr1KO generated by blastocyst-mediated transgenesis [30] and β-arrestin 1 flox/flox mice crossed with mice containing β-actin (ACTB)-cre recombinase [31] to delete β-arrestin 1 broadly in all tissues (Fig. 1a).

We measured contractile force of isolated EDL muscle from WT control and βarr1KO mice after chronic infusion of clenbuterol for 2 weeks. Isolated EDL muscle was suspended in an organ bath chamber supplemented with 20% oxygen to maintain skeletal muscle pO2 in the physiological range of 4 to 20 mmHg (0.5–2.5% O2) [32, 33]. In WT mice treated with clenbuterol, both specific twitch force and tetanic force normalized to CSA were augmented compared to vehicle-treated mice (Additional file 1: Table S1). In contrast, the clenbuterol induced enhancement of twitch, and tetanic forces in EDL muscle was completely abrogated in βarr1KO mice (Additional file 1: Table S1). We next tested the force-frequency effect of EDL muscle in vehicle and clenbuterol-treated mice. EDL muscle from WT mice treated with clenbuterol showed an enhanced force-frequency effect as indicated by an upward shift of the curve at low frequencies compared to vehicle-treated mice (*P* < 0.001 for strength of stimuli, *P* < 0.001 for drug treatment by two-way ANOVA) (Fig. 1b). Whereas EDL muscle isolated from clenbuterol treated βarr1KO mice did show an increase in force with stimulation frequency, the clenbuterol-induced enhancement of the force-frequency response was completely abolished to a level similar to vehicle treated mice (*P* < 0.01 by two-way ANOVA with Sidak’s multiple comparison test) (Fig. 1c). To test the role of β-arrestin 1 in fatigue development, we measured the time of maximum force generation to fall to 50% of the maximum response. In WT mice, fatigue development was shorter with clenbuterol treatment (66.4 ± 3.3 s) than with vehicle treatment (79.7 ± 3.9 s) indicating increased susceptibility to fatigue (*P* < 0.05 by Student’s *t* test) (Additional file 1: Table S1). The effect of clenbuterol-mediated susceptibility to fatigue was lost in the βarr1KO compared to WT mice (Additional file 1: Table S1). Taken together, these data indicate that β-arrestin 1 is essential in mediating the β_2_AR-stimulated enhancement in contractile properties of skeletal muscle as assessed by twitch, tetanus, force-frequency, and fatigue parameters.

### β-arrestin 1 mediates the β_2_AR stimulated increase in Ca^2+^ transients in skeletal muscle

**Fig. 2:**
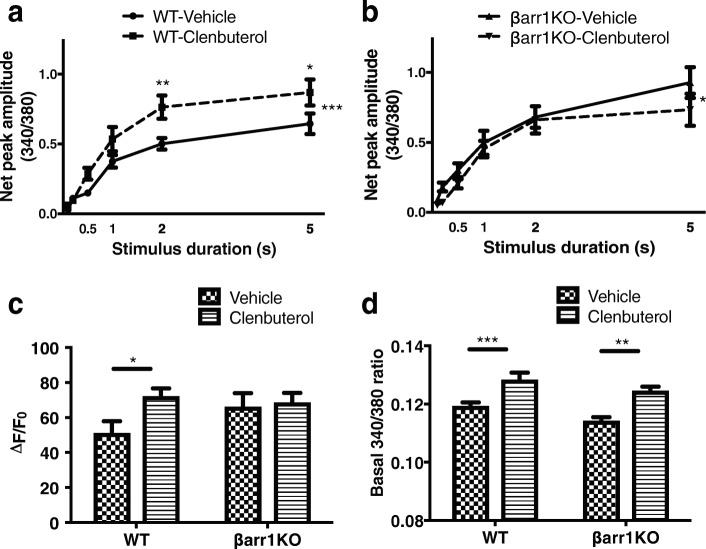
β-arrestin 1 is required for enhanced Ca^2+^ transient in FDB fiber upon β_2_AR activation. Net peak amplitude of Ca^2 +^ transient at different stimulation duration of FDB fibers after 2 weeks of stimulation with vehicle or clenbuterol (1 mg/kg/day) from WT (**a**) and βarr1KO (**b**). Data represent the mean ± SEM from at least five independent experiments. Statistical comparison of the curves was performed by using a two-way ANOVA with Sidak’s multiple comparison test (*, *P* < 0.05; ***, *P* < 0.001). Averaged peak fluorescence ratio ( ΔF/F_0_) (**c**) and basal transient (**d**) obtained from vehicle or clenbuterol (1 mg/kg/day) treated in WT and βarr1KO FDB fibers. Data are expressed as mean ± SEM from five independent experiments. Statistical comparison was performed by using a two-way ANOVA with Tukey’s multiple comparison test and student t-test (*, *P* < 0.05; **, *p* < 0.01; ***, *P* < 0.001)

In skeletal muscle, membrane depolarization triggers the release of Ca^2+^ ions from the sarcoplasmic reticulum to initiate muscle contraction. Mobilization of Ca^2+^ from intracellular stores can be enhanced through β_2_AR stimulation via canonical (protein kinase A and exchange protein directly activated by cAMP) [34] and non-canonical (phospholipase C and inositol trisphosphate receptor) pathways [35]. To test whether deletion of β-arrestin 1 would influence Ca^2+^ signaling secondary to β_2_AR activation, we conducted a series of calcium fluorescence imaging experiments on single muscle fibers isolated from FDB muscle of WT and βarr1KO mice. To evaluate the role of β-arrestin 1 on the Ca^2+^ response, we stimulated fibers for 0.1, 0.2, 0.5, 1, 2, and 5 s at 50 Hz using a higher affinity Ca^2+^ indicator, fura-4 AM. In WT FDB fibers, cytosolic Ca^2+^ levels were higher in clenbuterol-treated fibers compared to vehicle-treated fibers (Fig. 2a). In FDB fibers isolated from βarr1KO mice, clenbuterol treatment led to the loss of the stimulation-induced increase in cytosolic Ca^2+^ at 0.1-2 s and reversal at the 5 s stimulation time point (Fig. 2b). The average change from fluo-4 signals normalized to the amplitude of the initial Ca^2+^ transient (Δ*F*/*F*_0_) showed that clenbuterol treatment increased the amplitude of the Ca^2+^ transient compared to vehicle-treated WT fibers (51% for vehicle, 72% for clenbuterol, *P* < 0.01) (Fig. 2c). We also measured the basal level of Ca^2+^ release using fluorescent fura-2-loaded FDB fibers. After 2 weeks of chronic clenbuterol administration, resting cytosolic Ca^2+^ levels of FDB fibers isolated from WT and βarr1KO mice were similarly increased compared to vehicle administration (7% for WT, 9% for βarr1KO, *P* < 0.05) (Fig. 2d). The amplitudes of clenbuterol-treated fibers were suppressed in βarr1KO fibers, eliminating the clenbuterol effect on Ca^2+^ influx from prolonged stimuli. Therefore, β-arrestin 1 appears to modulate the clenbuterol-mediated release of intracellular Ca^2+^ stores in FDB fibers elicited by electrical stimulation.

### Increased skeletal muscle mass induced by clenbuterol is decreased by ablation of β-arrestin 1

**Fig. 3:**
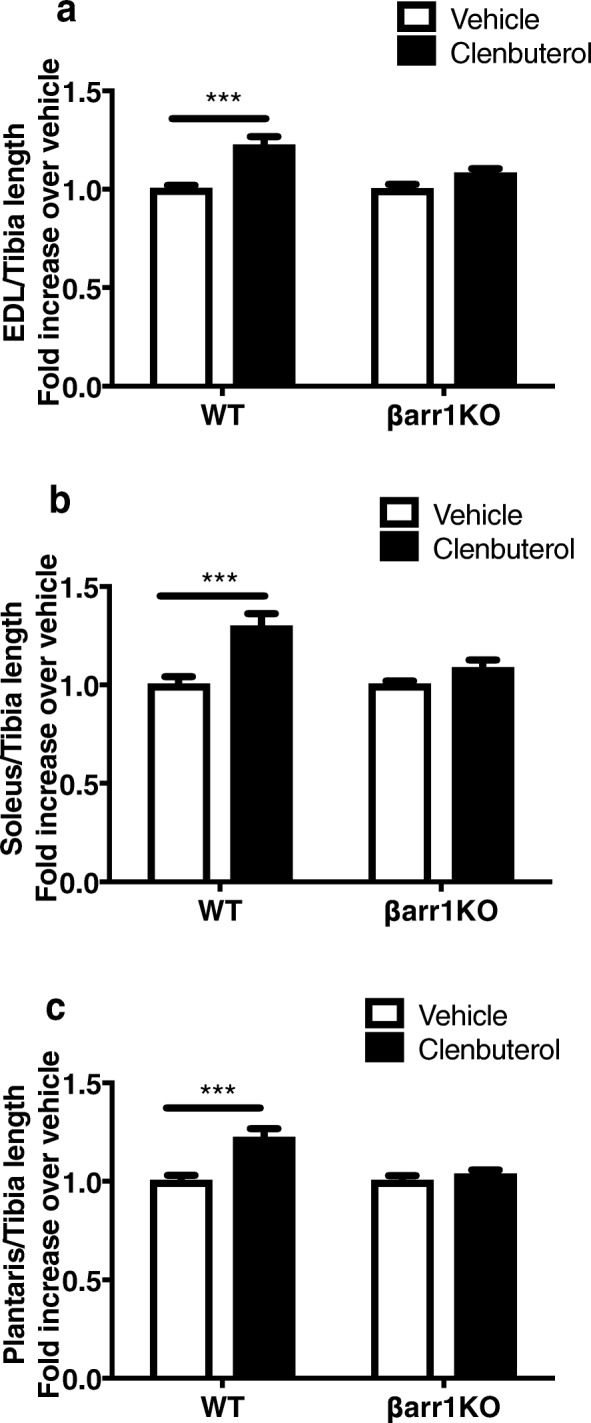
The increase in skeletal muscle mass induced by clenbuterol is eliminated by ablation of β-arrestin 1**.** Muscle weight normalized by tibia length for EDL (**a**), soleus (**b**) and plantaris (**c**) were present from WT and βarr1KO mice after 4 weeks treatment of vehicle or clenbuterol (1 mg/kg/day). Normalized muscle index was presented as a fold increase over vehicle stimulation. Means ± SEM are shown from 7 to 8 mice per group. Statistical comparison was performed by using a two-way ANOVA with Tukey’s multiple comparison test (***, *P* < 0.001)

Skeletal muscle is classified as fast- or slow-twitch muscle depending on their energy metabolism. EDL muscle, a typical fast-twitch muscle, largely utilizes glycolytic metabolism as the energy source, while the primarily slow-twitch soleus muscle mainly relies on oxidative metabolism [36]. The plantaris muscle utilizes both glycolytic and oxidative metabolism and adapts in response to physiological demands such as endurance exercise [37]. Due to these distinct characteristics, we examined the role of β-arrestin 1 in the β_2_AR-mediated hypertrophic response of these three muscles after chronic administration of clenbuterol (1 mg/kg/day) or vehicle for 4 weeks. We observed a 30 ± 6% increase of muscle weight in soleus muscle from WT after the chronic clenbuterol administration, as well as 22 ± 4% and 22 ± 5% increases in the mass of both EDL and plantaris, compared to the respective vehicle-treated muscles (*P* < 0.001 by two-way ANOVA with Tukey’s multiple comparison test) (Fig. 3, Additional file 2: Table S2). Though clenbuterol effects were previously reported to be fiber-type specific [38, 39], we observed that clenbuterol induced a similar hypertrophic response in all three muscle tissues. In contrast, clenbuterol administration to βarr1KO mice failed to increase muscle mass for any of the tissue types (Fig. 3, Additional file 2: Table S2). These data demonstrate the importance of β-arrestin 1 as an essential regulator of β_2_AR-mediated skeletal muscle hypertrophic growth.

### β-arrestin 1 is required for β_2_AR-mediated fiber size hypertrophy

**Fig. 4:**
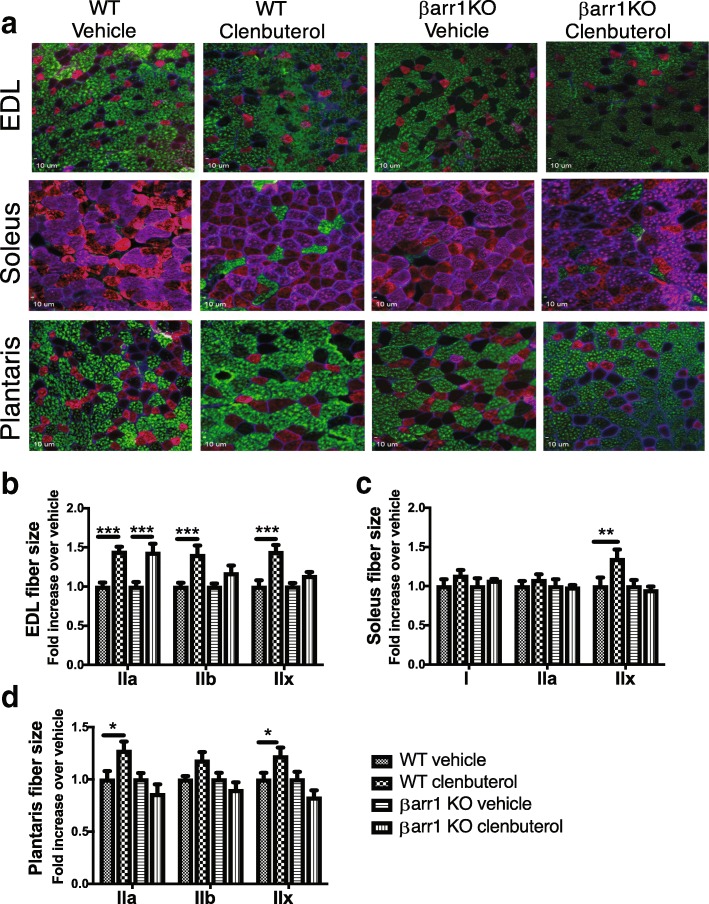
β-arrestin 1 is required for β2AR-mediated fiber size hypertrophy. (**a**) Representative immunohistochemistry images of MHC isoforms were presented from EDL, soleus, and plantaris muscles from WT and βarr1KO mice treated by vehicle or clenbuterol (1 mg/kg/day) for four weeks. Multicolor immunohistochemistry was performed with primary antibodies against MHC I (BA-F8), MHC IIa (SC-71, 2F7), MHC IIb (BF-F3), and polyclonal dystrophin. Staining was visualized simultaneously using Alexa Fluor 488 anti-mouse IgG2a (BF-F3, MHC IIb, Green), Alexa Fluor 568 anti-mouse IgG1 (SC-71, MHC IIa, Red), Alexa Fluor 647 anti-mouse IgG2b (BA-F8, MHC I, Purple), and Alexa Fluor 405 anti-rabbit (dystrophin, Blue). Fiber size of MHC I, IIa, IIb, and IIx was measured and presented as a fold increase over vehicle stimulation for EDL (**b**), soleus (**c**), and plantaris (**d**). Data shown represent the means ± SEM from 6 to 8 mice per group. Statistical comparison was performed by using a two-way ANOVA with Tukey’s multiple comparison test (*, *P* < 0.05; **, *p* < 0.01; ***, *P* < 0.001)

Clenbuterol is reported to induce cellular hypertrophy in fast-twitch type II skeletal muscle fibers and myotoxicity exclusively in slow twitch type I fibers [40]. We therefore analyzed fiber size and fiber composition of EDL, soleus, and plantaris muscles from WT and βarr1KO mice on histological cross-sections of muscles labeled with anti-dystrophin, BF-F3 (MHC IIB), Sc-71(MHC IIA), and BA-F8 (MHC I) (Fig. 4a). Chronic clenbuterol administration increased the size of all three types of fibers in fast-twitch EDL muscle (IIa by 45%, IIb by 41%, IIx by 44%, *P* < 0.001) (Fig. 4b). Ablation of β-arrestin 1 blunted the increase in clenbuterol-stimulated EDL fiber size in IIb and IIx fibers but not in IIa fibers (Fig. 4b). While clenbuterol had no effect on muscle fiber size among WT oxidative fibers (I and IIa) in the slow-twitching soleus muscle, IIx fibers showed a 35% increase in response to clenbuterol treatment. No hypertrophic response was observed for IIx fibers in βarr1KO mice (Fig. 4c). In the plantaris muscle, clenbuterol induced a modest increase in fiber size in WT muscle, which was again abrogated by deletion of β-arrestin 1 (Fig. 4d).

Since clenbuterol is known to induce a shift in muscle composition from slow oxidative to fast glycolytic fiber types [41, 42], we measured the abundance of type I slow-oxidative twitch fibers, fast-twitch fibers type IIa **(**fast oxidative fibers), and type IIb and IIx fibers (fast glycolytic fibers) in the three muscle groups (Table 1). Muscle composition of βarr1KO mice showed slight differences compared to their soleus and plantaris muscles of WT mice suggesting a baseline role of β-arrestin 1 in fiber type composition independent of exogenous β2AR stimulation (Table 1). Clenbuterol treatment increased the percentage of fast-twitch IIb fibers in all tissue types. Notably, we observed similar clenbuterol-induced percentage increases in IIb fibers in all tissue types in the βarr1KO mice as well suggesting β-arrestin 1 does not play a significant role in clenbuterol-mediated increases in IIb muscle fibers. Taken together, these data show that β-arrestin 1 differentially modulates muscle cell growth in response to β_2_AR stimulation with little effect on fiber type remodeling as assessed by fiber composition.

**Table 1:** Effect of clenbuterol on skeletal muscle fiber composition of EDL

	WT		βarr1KO		*P* value	
	Vehicle	Clenbuterol	Vehicle	Clenbuterol	Drug	Strain
EDL						
I (%)	0.0 ± 0.0	0.1 ± 0.1	0.3 ± 0.2	0.9 ± 0.5		
IIa (%)	12.7 ± 1.6	11.4 ± 1.4	13.7 ± 1.3	9.6 ± 1.7 *		
IIb (%)	59.2 ± 3.7	68.4 ± 3.3 *	65.2 ± 2.3	73.7 ± 2.8 **	*	
IIx (%)	28.1 ± 4.9	20.1 ± 2.1 **	20.8 ± 1.9	15.8 ± 2.2 *	*	
I + IIa (%)	12.7 ± 1.6	11.5 ± 1.2	14.0 ± 1.4	10.5 ± 1.3 *		
IIb + IIx (%)	87.3 ± 1.6	88.5 ± 1.2	86.0 ± 1.4	89.5 ± 1.3 *		
N	7	8	7	8		
Soleus						
I (%)	48.8 ± 1.4	44.2 ± 1.8	48.9 ± 3.6	51.0 ± 2.0		
IIa (%)	44.6 ± 1.6	44.5 ± 1.4	42.8 ± 3.3	36.6 ± 1.7		*
IIb (%)	0.0 ± 0.0	2.5 ± 0.8 *	1.3 ± 0.8	5.0 ± 1.2 *	**	*
IIx (%)	6.7 ± 1.1	8.8 ± 0.9	7.0 ± 1.2	7.4 ± 1.2		
I + IIa (%)	93.3 ± 1.1	88.8 ± 1.1 *	91.8 ± 1.3	87.6 ± 1.1 *	**	
IIb + IIx (%)	6.7 ± 1.1	11.3 ± 1.1 *	8.2 ± 1.3	12.4 ± 1.1*	**	
N	6	8	7	8		
Plantaris						
I (%)	0.0 ± 0.0	0.5 ± 1.2	2.9 ± 0.6	1.2 ± 0.3		*
IIa (%)	33.3 ± 2.2	25.8 ± 2.0 *	23.7 ± 3.3	20.2 ± 0.8	*	**
IIb (%)	48.2 ± 3.7	58.3 ± 3.2 *	52.2 ± 4.7	68.5 ± 1.3 **	***	*
IIx (%)	18.6 ± 2.3	15.1 ± 2.6	21.2 ± 1.5	10.2 ± 1.0 **	**	
I + IIa (%)	33.3 ± 2.4	26.2 ± 2.0 *	26.6 ± 3.5	21.4 ± 0.8	*	*
IIb + IIx (%)	66.7 ± 2.4	73.8 ± 2.0 *	73.4 ± 3.5	78.6 ± 0.8	*	*
N	7	8	7	8		

Values are means ± SEM. *N*, the number of muscles utilized for the analysis. *P* value next to the numbers compared to vehicle treatment. *P* values for drug and strain were calculated by a two-way ANOVA with Tukey’s multiple comparison test. *P < 0.05, **P < 0.01, ***P < 0.001

### Clenbuterol-mediated phosphorylation events are β-arrestin 1 dependent in primary cultured myoblasts

**Fig. 5:**
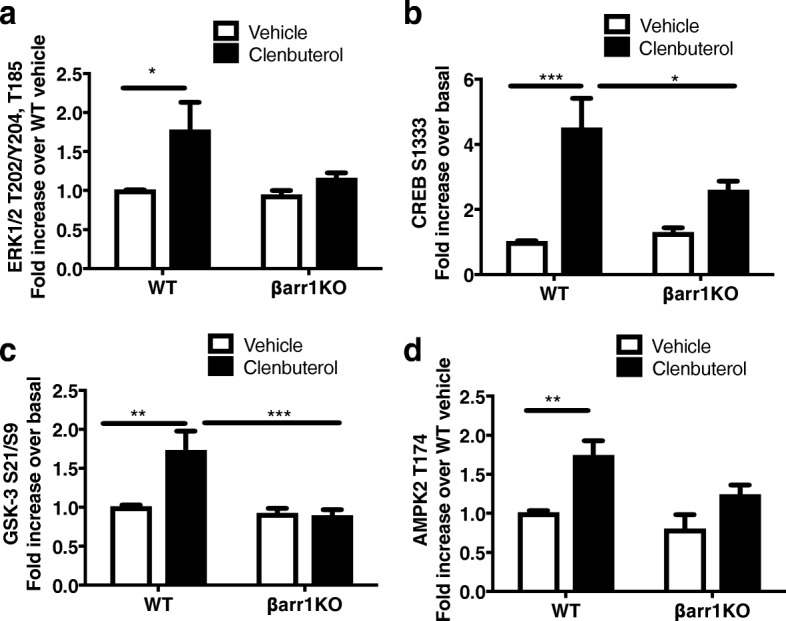
Human phospho-antibody array analysis reveals that phosphorylation events induced by clenbuterol in primary myoblasts are primarily β-arrestin 1–dependent. Normalized intensities for four representative phosphoproteins of ERK (**a**), CREB (**b**), GSK3 (**c**), and AMPK2 (**d**) applied to the phospho-antibody array. Lysates were from primary myoblasts isolated from WT and βarr1KO followed by 10 μM clenbuterol stimulation for 5 min or no stimulation (vehicle). The normalized intensity for each antibody was calculated as a fold increase over vehicle stimulation of primary myoblast from WT mice. Data shown represent the means ± SEM from five independent experiments. Statistical analysis was performed using a two-way ANOVA with Tukey’s multiple comparison test (*P < 0.05; **P < 0.01; ***P < 0.001)

To address the potential cellular mechanisms for β-arrestin 1-mediated signaling in skeletal muscle, we performed a targeted phosphoproteomic analysis on clenbuterol stimulated primary cultured myoblasts from WT and βarr1KO mice. We detected 35 phosphoproteins that were elevated in clenbuterol-treated WT myoblasts, whereas we observed fewer changes in phosphoprotein stimulation in clenbuterol-treated myoblasts from βarr1KO mice (Additional file 3: Table S3). Of the signaling pathways known to be β-arrestin 1 dependent [14, 25], we found that clenbuterol led to a 1.8-fold increase in extracellular-regulated kinase (ERK) phosphorylation in WT myoblasts that was abolished in myoblasts from βarr1KO mice (Fig. 5a). Similarly, clenbuterol led to activation of other known hypertrophic signaling molecules in WT myoblasts such as p38, c-Jun N-terminal kinase (JNK), P70S6 kinase, ribosomal S6 kinase (RSK), and mitogen-activated protein kinase kinase (MEK), which were all blunted in clenbuterol-treated myoblasts from βarr1KO mice (Additional file 3: Table S3). In myoblasts obtained from WT mice, clenbuterol-induced robust phosphorylation of critical downstream targets of β_2_AR stimulation, such as cAMP response element binding protein (CREB) (Fig. 5b), glycogen synthase kinase (GSK)-3 (Fig. 5c), and 5′AMP-activated protein kinase (AMPK) 2 (Fig. 5d), all of which were prevented with β-arrestin 1 depletion (Additional file 3: Table S3). Overall, we observed that a broad range of protein kinases in skeletal myoblasts require β-arrestin 1 for their activation in response to β_2_AR stimulation.

## Discussion

In this study, we explored the role of β-arrestin 1 in skeletal muscle remodeling and performance in response to chronic β_2_AR agonist administration. β_2_AR activation by administration of clenbuterol enhanced the contractile properties of fast-twitch EDL muscle fibers and augmented the Ca^2+^ transient amplitude of FDB fibers; responses that were abolished in muscle lacking β-arrestin 1. Moreover, the increase in skeletal muscle fiber size with chronic clenbuterol-induced β_2_AR stimulation was β-arrestin 1-dependent indicating that β-arrestin 1 is a critical signaling molecule regulating skeletal muscle growth and function downstream of the β_2_AR. Our findings support the concept that enhancing β-arrestin 1-dependent, β_2_AR-mediated signaling pathways may be useful in stimulating muscle growth in conditions of muscle wasting including the elderly and patients suffering from chronic illness such as cancer or heart failure.

Skeletal muscle wasting is a significant medical problem, particularly in individuals vulnerable to the catabolic effects of muscle atrophy and age-related diseases. Anabolic androgenic steroids have been used to treat muscle wasting associated with surgery and cancer, as they can increase muscle mass, myofiber size, and number of satellite cells in skeletal muscle [43, 44]. However, the side effects of long-term use of anabolic steroids have limited their widespread use [45]. The use of insulin-like growth factor 1 (IGF-1) has also been investigated as it plays a major role in the regulation of skeletal muscle growth [46, 47]. While studies have shown improved physical performance, side effects such as hypoglycemia and risk for the development of cancer have limited the use of IGF-1 for this indication [48].

In this study, we found that enhanced skeletal muscle growth and contractility by clenbuterol treatment required β-arrestin 1 in skeletal muscle. β-arrestins have previously been shown to promote cardiomyocyte [19] and uterine contractility [49]. For instance, a β-arrestin-biased pepducin of the β_2_AR was recently shown to induce cardiomyocyte contractility and antiapoptotic signaling [50], whereas β-arrestin functioning as a scaffold for Ca^2+^/calmodulin-dependent protein kinase II (CaMKII) activity and exchange protein directly activated by cAMP (Epac) was detrimental to cardiac function following myocardial infarction [51, 52]. Additionally, β-arrestin 2, but not by β-arrestin 1, interacts with sarcoendoplasmic reticulum calcium transport ATPase (SERCA) 2a to enhance cardiomyocyte contractility [53]. Taken together, these studies highlight the role of β-arrestins in modulating muscle physiology by linking receptor activation to downstream effectors by assembling multimeric complexes to initiate signaling.

Our data show that clenbuterol stimulated Ca^2+^ transients from βarr1KO fibers were markedly blunted indicating a dependence on β-arrestin 1 signaling for excitation-contraction (EC) coupling in skeletal muscle. EC coupling involves a series of steps that link membrane depolarization with Ca^2+^ release from the sarcoplasmic reticulum (SR) to generate the cytosolic Ca^2+^ transient followed by the resequestration of Ca^2+^ back into the SR to restore the Ca^2+^ transient to resting levels. The mechanism of action for how β-arrestin 1 modulates E-C coupling may occur at a number of levels such as changes in ryanodine receptor 1 (RYR1)-Ca^2+^ release, SERCA1 pumping activity, sodium-calcium exchanger 1 activity, or altered Ca^2+^ buffering capacity.

From our Ca^2+^ imaging studies, we show that chronic clenbuterol administration raises resting cytosolic Ca^2+^ levels that is independent of β-arrestin 1 signaling since Ca^2+^ levels were increased in fibers from both WT and βarr1KO mice. Though changes in phospholamban phosphorylation or SERCA1 activity might create an imbalance between SR Ca^2+^ uptake and release, the increase in cytosolic Ca^2+^ resulting from a SR Ca^2+^ imbalance (RYR leak versus SERCA1 reduced capacity) would need to come from a change in the rates of flux (reduced extrusion or increase entry) across the sarcolemma in order to effect change in resting cytosolic Ca^2+^ levels [54]. While our data supports a concept that β-arrestin 1 functions to modulate the EC coupling process to alter Ca^2+^ influx in skeletal muscle, additional studies will be needed to fully appreciate the complex mechanisms by which β-arrestin 1 acts to augment the clenbuterol stimulated Ca^2+^ transient.

The selective β_2_AR agonist clenbuterol, approved for use as a bronchodilator outside the USA, can increase muscle weight in experimental animal models [55], and therefore has been suggested as a better therapeutic option over steroids or IGF-1 for muscle wasting [3, 56]. The potential use of clenbuterol to counteract muscle wasting has been studied in a number of pathological conditions such as that induced by the glucocorticoid dexamethasone [57], Pompe’s disease [58, 59], heart failure [60], and murine models of Duchenne’s muscular dystrophy [61, 62]. Administration of β_2_AR agonists results in a pronounced shift in the skeletal muscle fiber type profile from slow-oxidative to fast-glycolytic [42, 63] and may account, in part, for the mechanism of its salutary effect in sarcopenia, where most age-related atrophy occurs in fast-twitch fibers that are important for high-intensity anaerobic movements [10, 64, 65]. Our data add to this literature by demonstrating that β_2_AR-induced skeletal muscle fiber hypertrophy and enhanced performance is β-arrestin 1-dependent, while changes in fiber type (slow to fast) do not require β-arrestin 1 and are likely G-protein dependent.

We observed that a broad range of protein kinases in skeletal myoblasts require β-arrestin 1 for their activation in response to β_2_AR stimulation. Indeed, it has been shown for the β_1_AR that isoproterenol stimulation leads to the formation of receptor-β-arrestin complexes to promote intracellular ERK signaling [66]. ERK1/2 and related downstream pathways have been shown to be downstream effectors of β-arrestin-dependent signaling pathways upon GPCR stimulation [25, 67], consistent with our findings in skeletal myoblasts. β-arrestins have been shown to interact with proteins involved in the regulation of apoptosis such as GSK3β, heat shock protein 27, apoptosis signal-regulating kinase 1, and Bcl2-promoted death promotor, decreasing rate of apoptosis [14]. While identifying the precise mechanisms by which β-arrestins regulate downstream signaling effectors in the skeletal muscle will require additional study, our data provide evidence for the importance of β-arrestins on skeletal muscle function during the progression of pathologic states such as muscle wasting.

The concept of GPCR signaling bias or functionally selective signaling is providing the framework for the development of a new class of pharmacological agents where a ligand stimulates a subset of a given receptor’s signal transducers with the potential for reduced side effects over their unbiased counterparts [68]. Prior work in other organ systems has highlighted an important role for β-arrestin 1 in β_2_AR signaling in several pathological conditions in vivo [19, 69]. Since the roles of β-arrestin 1 in regulating β_2_AR signaling differ among pathological conditions, a better understanding of the mechanisms by which β_2_AR activation leads to enhanced muscle performance may provide a platform to discover drugs that selectively activate β-arrestin 1 signaling as therapeutic candidates for pathological conditions associated with skeletal muscle wasting.

## Conclusions

We have identified a previously unappreciated role for β-arrestin 1 in mediating β_2_AR-stimulated skeletal muscle growth and strength. We propose these findings could have important implications in the design of future pharmacologic agents aimed at reversing pathological conditions associated with skeletal muscle wasting.

## Additional files

Additional file 1: **Table S1.** The effect of clenbuterol on EDL muscle contraction. (DOCX 82 kb)

Additional file 2: **Table S2.** Clenbuterol-mediated skeletal muscle hypertrophy in WT and βarr1KO mice. (DOCX 87 kb)

Additional file 3: **Table S3.** A list of β-arrestin 1-regulated phosphoproteins revealed by human phospho-antibody array analysis. (DOCX 155 kb)

## Abbreviations

AMPK: 5′ AMP-activated protein kinase
BSA: Bovine serum albumin
Ca^2+^: Calcium
cAMP: Cyclic adenosine monophosphate
CREB: cAMP response element binding
CSA: Cross-section area
DMEM: Dulbecco’s modified Eagle’s medium
EC: Excitation contraction coupling
EDL: Extensor digitorum longus
ERK: Extracellular-regulated kinase
FDB: Flexor digitorum brevis
GAPDH: Glyceraldehyde 3-phosphate dehydrogenase
GPCR: G protein-coupled receptor
GRK: G protein-coupled receptor kinase
GSK: Glycogen synthase kinase
IGF1: Insulin-like growth factor-1
JNK: c-Jun N-terminal kinase
MEK: Mitogen activated protein kinase kinase
MHC: Myosin heavy chain
RSK: Ribosomal S6 kinase
RYR: Ryanodine receptor
SEM: Standard error of the mean
SERCA: The sarcoendoplasmic reticulum calcium transport ATPase
SR: Sarcoplasmic reticulum
WT: Wild type
β_2_AR: β_2_ adrenergic receptor
βarr1KO: β-arrestin 1 knockout
