# Supplementary material for: β-arrestin 1 regulates β2-adrenergic receptor-mediated skeletal muscle hypertrophy and contractility
Source: Skelet Muscle. 2018 Dec 27;8:39. doi: 10.1186/s13395-018-0184-8 (PMC6309084; doi:10.1186/s13395-018-0184-8)
Supplement: Supplementary file 1 — Table S1. The effect of clenbuterol on EDL muscle contraction. (DOCX 82 kb) [file 13395_2018_184_MOESM1_ESM.docx]

Table S1. **The effect of clenbuterol on EDL muscle contraction**

|  | **WT** | | **βarr1KO** | |
| --- | --- | --- | --- | --- |
|  | **DMSO** | **Clenbuterol** | **DMSO** | **Clenbuterol** |
| **Twitch force, mN** | 38.5 ± 6 (7) | 56.5 ± 4.5 * (7) | 46.5 ± 6.9 (8) | 38.6 ± 6.6 (8) |
| **Twitch force/CSA, mN/mm2** | 46.7 ± 7.6 (7) | 60.2 ± 7.4 * (7) | 59.7 ± 5.8 (8) | 39.3 ± 5.2(8) |
| **Tetanic force, mN** | 145.7± 17.6 (7) | 196.5 ± 16.7 (6) | 172.6 ± 23.3 (8) | 125.8 ± 30.3 (7) |
| **Tetanic force/CSA, mN/mm2** | 176.1 ± 23.4 (7) | 214.2 ± 25.9* (6) | 225.6 ± 19 (8) | 126.9 ± 25.3* (7) |
| **Twitch-to-tetanic force ratio** | 0.3 ± 0.0 (7) | 0.3 ± 0.0 (6) | 0.3 ± 0.0 (8) | 0.3 ± 0.0 (7) |
| **Fatigue (time to 50% of maximum), sec** | 79.7 ± 3.9 (7) | 66.4 ± 3.3* (5) | 68.1 ± 5.2 (7) | 75.2 ± 6.4 (5) |

Values are means ± SEM. The number of muscles utilized for the analysis was indicated in parenthesis. Twitch and tentanic forces are adjusted by muscle fiber cross sectional area (CSA). N, the number of muscles utilized for the analysis in parenthesis. * P < 0.05 compared to its vehicle treatment by unpaired student t-test.
