# Supplementary material for: β-arrestin 1 regulates β2-adrenergic receptor-mediated skeletal muscle hypertrophy and contractility
Source: Skelet Muscle. 2018 Dec 27;8:39. doi: 10.1186/s13395-018-0184-8 (PMC6309084; doi:10.1186/s13395-018-0184-8)
Supplement: Supplementary file 2 — Table S2. Clenbuterol-mediated skeletal muscle hypertrophy in WT and βarr1KO mice. (DOCX 87 kb) [file 13395_2018_184_MOESM2_ESM.docx]

**Table S2.** **Clenbuterol-mediated skeletal muscle hypertrophy in WT and βarr1KO mice**

|  | **WT** | | **βarr1KO** | |
| --- | --- | --- | --- | --- |
|  | **Vehicle** | **Clenbuterol** | **Vehicle** | **Clenbuterol** |
| **Body weight (g)** | 22.57 ± 0.45 | 24.06 ± 0.63 | 25.62 ± 0.66 | 25.94 ± 0.23 |
| **Body length (cm)** | 8.89 ± 0.14 | 9.01 ± 0.08 | 9.81 ± 0.07 | 9.69 ± 0.04 |
| **Tibia length (TL)(cm)** | 1.64 ± 0.02 | 1.57 ± 0.04 | 1.75 ± 0.02 | 1.76 ± 0.01 |
| **EDL (mg)** | 6.94 ± 0.18 | 8.03 ± 0.25 | 7.98 ± 0.22 | 8.06 ± 0.25 |
| **Soleus (mg)** | 6.03 ± 0.29 | 7.41 ± 0.37 | 7.13 ± 0.18 | 7.26 ± 0.37 |
| **Planteris (mg)** | 12.18 ± 0.45 | 14.08 ± 0.54 | 14.64 ± 0.48 | 14.19 ± 0.56 |
| **EDL/TL (mg/cm)** | 4.22 ± 0.08 | 5.14 ± 0.19 *** | 4.56 ± 0.12 | 4.93 ± 0.11 |
| **Soleus /TL (mg/cm)** | 3.65 ± 0.15 | 4.73 ± 0.24 *** | 4.07 ± 0.08 | 4.41 ± 0.17 |
| **Planteris /TL (mg/cm)** | 7.38 ± 0.22 | 9.00 ± 0.35 *** | 8.36 ± 0.24 | 8.64 ± 0.20 |
| **N** | 8 | 8 | 7 | 8 |

Values are means ± SE. N, the number of muscles utilized for the analysis. ***, P < 0.001 compared to its vehicle treatment by two-way ANOVA with Turkey’s multiple comparison test.
