# Supplementary material for: β-arrestin 1 regulates β2-adrenergic receptor-mediated skeletal muscle hypertrophy and contractility
Source: Skelet Muscle. 2018 Dec 27;8:39. doi: 10.1186/s13395-018-0184-8 (PMC6309084; doi:10.1186/s13395-018-0184-8)
Supplement: Supplementary file 3 — Table S3. A list of β-arrestin 1-regulated phosphoproteins revealed by human phospho-antibody array analysis. (DOCX 155 kb) [file 13395_2018_184_MOESM3_ESM.docx]

**Table S3. A list of β-arrestin 1-regulated phosphoproteins revealed by human phospho-antibody array analysis.**

|  | **WT** | | | **βarr1KO** | | |
| --- | --- | --- | --- | --- | --- | --- |
|  | **Vehicle** | **Clenbuterol** | **P-value** | **Vehicle** | **Clenbuterol** | **P-value** |
| **p27 T157** | 1.0 ± 0.1 | 6.6 ± 1.5 | *** | 2.4 ± 0.5 | 1.1 ± 0.4 | * |
| **p53 S46** | 1.0 ± 0.0 | 4.9 ± 0.9 | *** | 1.4 ± 0.5 | 1.5 ± 0.3 |  |
| **CREB S133** | 1.0 ± 0.3 | 4.5 ± 0.9 | ** | 1.3 ± 0.2 | 2.6 ± 0.3 | *** |
| **Lyn Y397** | 1.0 ± 0.1 | 4.4 ± 1.5 | ** | 1.0 ± 0.2 | 1.5 ± 0.5 |  |
| **p27 T198** | 1.0 ± 0.1 | 4.1 ± 1.1 | ** | 2.0 ± 0.5 | 1.3 ± 0.5 |  |
| **p53 S392** | 1.0 ± 0.1 | 3.9 ± 1.2 | * | 1.5 ± 0.1 | 1.2 ± 0.1 |  |
| **Lck Y394** | 1.0 ± 0.1 | 3.6 ± 1.0 | * | 0.8 ± 0.2 | 1.2 ± 0.1 |  |
| **STAT3 Y705** | 1.0 ± 0.1 | 3.4 ± 1.1 | * | 0.5 ± 0.2 | 1.4 ± 0.3 | *** |
| **Chk-2 T68** | 1.0 ± 0.0 | 3.3 ± 0.8 | ** | 1.0 ± 0.3 | 1.3 ± 0.2 |  |
| **RSK1/2/3 S380/S386/S377** | 1.0 ± 0.0 | 3.0 ± 0.7 | ** | 1.0 ± 0.3 | 1.1 ± 0.3 |  |
| **FAK Y397** | 1.0 ± 0.0 | 3.0 ± 1.0 | * | 1.3 ± 0.3 | 0.9 ± 0.3 |  |
| **Yes Y426** | 1.0 ± 0.1 | 2.9 ± 0.5 | ** | 1.5 ± 0.5 | 1.6 ± 0.2 |  |
| **p53 S15** | 1.0 ± 0.1 | 2.8 ± 0.9 | * | 1.0 ± 0.2 | 1.0 ± 0.4 |  |
| **Src Y419** | 1.0 ± 0.1 | 2.7 ± 0.5 | * | 1.3 ± 0.1 | 1.7 ± 0.1 |  |
| **MEK1/2 S218/S222, S222/S226** | 1.0 ± 0.1 | 2.6 ± 0.6 | * | 1.3 ± 0.3 | 1.6 ± 0.2 |  |
| **Fyn Y420** | 1.0 ± 0.1 | 2.6 ± 0.4 | *** | 1.3 ± 0.1 | 1.7 ± 0.1 |  |
| **AMPK1 T174** | 1.0 ± 0.1 | 2.6 ± 0.8 | * | 0.9 ± 0.1 | 1.3 ± 0.2 | * |
| **p70 S6 Kinase T389** | 1.0 ± 0.1 | 2.5 ± 0.7 | * | 1.0 ± 0.1 | 0.8 ± 0.1 |  |
| **p38 T180/Y182** | 1.0 ± 0.1 | 2.5 ± 0.4 | *** | 1.3 ± 0.1 | 1.6 ± 0.3 |  |
| **p70 S6 Kinase T421/S424** | 1.0 ± 0.2 | 2.5 ± 0.4 | *** | 0.9 ± 0.2 | 1.1 ± 0.2 |  |
| **Hck Y411** | 1.0 ± 0.1 | 2.3 ± 0.7 | * | 1.1 ± 0.2 | 1.4 ± 0.2 |  |
| **HSP27 S78/S82** | 1.0 ± 0.1 | 2.2 ± 0.4 | * | 0.8 ± 0.2 | 1.3 ± 0.3 | * |
| **Akt T308** | 1.0 ± 0.1 | 2.2 ± 0.6 | * | 1.1 ± 0.2 | 0.9 ± 0.2 |  |
| **STAT6 Y641** | 1.0 ± 0.0 | 2.0 ± 0.3 | *** | 1.1 ± 0.2 | 1.2 ± 0.1 |  |
| **TOR S2448** | 1.0 ± 0.0 | 2.0 ± 0.4 | * | 1.1 ± 0.2 | 1.2 ± 0.2 |  |
| **Akt S473** | 1.0 ± 0.1 | 1.9 ± 0.5 | ** | 0.7 ± 0.2 | 0.7 ± 0.2 |  |
| **STAT5a/b Y694/Y699** | 1.0 ± 0.1 | 1.9 ± 0.2 | *** | 0.7 ± 0.0 | 0.8 ± 0.2 |  |
| **MSK1/2 S376/S360** | 1.0 ± 0.0 | 1.8 ± 0.3 | ** | 1.0 ± 0.1 | 1.3 ± 0.1 | * |
| **ERK1/2 T202/Y204, T185/Y187** | 1.0 ± 0.1 | 1.8 ± 0.4 | * | 0.9 ± 0.1 | 1.2 ± 0.1 | * |
| **AMPK2 T172** | 1.0 ± 0.0 | 1.7 ± 0.2 | ** | 0.8 ± 0.2 | 1.2 ± 0.1 | * |
| **GSK-3/ S21/S9** | 1.0 ± 0.0 | 1.7 ± 0.3 | * | 0.9 ± 0.1 | 0.9 ± 0.1 |  |
| **STAT2 Y689** | 1.0 ± 0.0 | 1.6 ± 0.3 | * | 1.1 ± 0.2 | 1.2 ± 0.1 |  |
| **STAT5a Y694** | 1.0 ± 0.1 | 1.6 ± 0.2 | *** | 0.7 ± 0.2 | 0.8 ± 0.1 |  |
| **β−Catenin** | 1.0 ± 0.1 | 1.5 ± 0.3 | ** | 0.5 ± 0.1 | 0.7 ± 0.1 | * |
| **JNK pan T183/Y185, T221/Y223** | 1.0 ± 0.1 | 1.5 ± 0.2 | ** | 0.7 ± 0.1 | 1.1 ± 0.1 | * |

Data are the mean ± SE of at least five experiments performed. The mean was calculated as a fold increase over vehicle stimulation of primary myoblast from WT. * Significantly different from its vehicle treatment, * P < 0.05, ** P < 0.01, *** P < 0.001.
